# Pathways to mental health services for young people: a systematic review

**DOI:** 10.1007/s00127-018-1578-y

**Published:** 2018-08-22

**Authors:** Kathleen MacDonald, Nina Fainman-Adelman, Kelly K. Anderson, Srividya N. Iyer

**Affiliations:** 1 Department of Psychiatry, McGill University, Montreal, QC Canada; 2 Prevention and Early Intervention Program for Psychosis (PEPP) and ACCESS Open Minds (pan-Canadian youth mental health services research network), Douglas Mental Health University Institute, Montreal, QC Canada; 3 Department of Epidemiology and Biostatistics, The University of Western Ontario, London, ON Canada; 4 Department of Psychiatry, The University of Western Ontario, London, ON Canada

**Keywords:** Youth mental health, Mental health services, Pathways to care, Help-seeking behaviour, Treatment delays

## Abstract

**Purpose:**

While early access to appropriate care can minimise the sequelae of mental illnesses, little is known about how youths come to access mental healthcare. We therefore conducted a systematic review to synthesise literature on the pathways to care of youths across a range of mental health problems.

**Methods:**

Studies were identified through searches of electronic databases (MEDLINE, PsycINFO, Embase, HealthSTAR and CINAHL), supplemented by backward and forward mapping and hand searching. We included studies on the pathways to mental healthcare of individuals aged 11–30 years. Two reviewers independently screened articles and extracted data.

**Results:**

Forty-five studies from 26 countries met eligibility criteria. The majority of these studies were from settings that offered services for the early stages of psychosis, and others included inpatient and outpatient settings targeting wide-ranging mental health problems. Generally, youths’ pathways to mental healthcare were complex, involved diverse contacts, and, sometimes, undue treatment delays. Across contexts, family/carers, general practitioners and emergency rooms featured prominently in care pathways. There was little standardization in the measurement of pathways.

**Conclusions:**

Except in psychosis, youths’ pathways to mental healthcare remain understudied. Pathways to care research may need to be reconceptualised to account for the often transient and overlapping nature of youth mental health presentations, and the possibility that what constitutes optimal care may vary. Despite these complexities, additional research, using standardized methodology, can yield a greater understanding of the help-seeking behaviours of youths and those acting on their behalf; service responses to help-seeking; and the determinants of pathways. This understanding is critical to inform ongoing initatives to transform youth mental healthcare.

**Electronic supplementary material:**

The online version of this article (10.1007/s00127-018-1578-y) contains supplementary material, which is available to authorized users.

## Introduction

Most psychiatric conditions emerge before the age of 25 [1]. Mental illness is the largest contributor to the burden of disability-adjusted life years (DALYs) among young people aged 0–24 in high-income countries and the seventh-highest contributor to DALYs in low- and middle-income countries. Globally, mental illnesses account for a quarter of all years lived with disability (YLDs) in children and youth aged 0–24 [2].

Despite this heavy burden, many youths with mental health problems remain untreated or face delayed detection, long waitlists and multiple help-seeking contacts before obtaining appropriate care [1, 3]. Such complex ‘pathways to care’ delay treatment. For youths (typically understood as individuals who are within the critical development juncture between childhood and adulthood, i.e., aged between 11 and 25–30 years old [4, 5]), longer durations of untreated illness can have grave impacts on the foundations of their adult lives and can be associated with worse clinical outcomes [6, 7].

Pathways to care—defined as the “sequence of contacts with individuals and organizations prompted by the distressed person’s efforts, and those of his or her significant others to seek help, as well as the help that is supplied in response to such efforts” [8]—have been garnering research attention for several years. In the early 1990s, a multinational study by the World Health Organization (WHO) [9] showed that pathways to mental healthcare varied substantially depending on context and resource availability. In regions with access to relatively well-developed mental health services, patients experienced more direct routes from the community to specialized care. However, in areas with few services, patients experienced a wide variety of pathways that often included traditional or faith healers.

In the field of first-episode psychosis, concern with the adverse consequences of delayed treatment [10] has spurred numerous investigations of pathways to care and barriers to accessing specialized services [11]. In addition to primary care providers and mental health services, help-seeking pathways for psychotic disorders involve diverse contacts like emergency rooms (ERs), social services, the criminal justice system, school counsellors, and religious agencies. Pathways to psychosis services have been known to be influenced by several sociodemographic factors, including gender, age, ethnicity, and socioeconomic status [12]. However, these findings have been inconsistent and their implications for policy and service delivery difficult to assess.

Sequences of healthcare contacts do not occur randomly [8], but are influenced by multiple intersecting individual, social, cultural, and systemic factors. Studying pathways to care allows us to identify the loci of barriers and delays to treatment; and key agents in the help-seeking process, including individuals in distress, family/carers, informal contacts (e.g., teachers, employers, web resources, etc.), and formal health services. Such knowledge is crucial for providing timely access to services.

New youth mental health initiatives [13], including in but not limited to Australia, Canada, Ireland and the United Kingdom, are striving to make appropriate services accessible early in the course of mental illnesses to mitigate their short- and long-term negative consequences. It has been argued that extant conventional mental health systems are neither youth-friendly nor sufficiently accessible. Young people and their families have described mental health help-seeking as a long, painful, and complicated journey. Though they represent the peak incidence of mental health problems, youths are frequently the least likely to use mental health services [14] and often receive help only when their problems become crises. Their help-seeking efforts may also be impeded by repeated evaluations and difficult transitions, especially between child and adult services [15].

Although literature reviews on pathways to care have been conducted in the field of psychosis [11, 16] and across adult mental health disorders [17], evidence on the different trajectories youths follow to obtain mental healthcare has yet to be synthesized. Such a synthesis is essential if efforts to transform youth mental healthcare [13, 18] are to achieve their ends. Our objective was therefore to conduct a systematic review of literature on young people’s pathways to care for a range of mental health problems.

## Methodology

The protocol for this systematic review was developed in accordance with the Preferred Reporting Items for Systematic reviews and Meta-Analyses [19] (PRISMA) and was registered at the PROSPERO Centre for Reviews and Dissemination (ID: 42016039208) in June 2016.

### Search strategy

Search terms were generated by consulting 20 experts in youth mental health across disorders, and a university librarian. We included search terms related to pathways to care; service utilization; help-seeking; mental disorders; and delays to treatment (see online supplementary material for search strategy).

Relevant studies were identified through searching five electronic databases: MEDLINE (1946 onward), Embase (1947 onward), PsycINFO (1967 onward), HealthSTAR (1966 onward) and CINAHL (1937 onward). Articles were further identified using backward and forward citation mapping of selected articles using Web of Science, and hand searches of journals that had previously published material on pathways (*n* = 4). The electronic search was conducted in July 2016 and updated in March 2018.

### Selection of relevant studies

Two experts independently screened titles, abstracts, and keywords and resolved disagreements by consensus. Articles were included if they were peer-reviewed; were written in English or French; and reported quantitative findings. To be selected, studies had to focus on youths’ individual trajectories to seeking or receiving treatment for mental health or substance use at any establishment, regardless of the presence or absence of a formal diagnosis. The mean age of study participants had to be between 11 and 30 years (so as to include the largest possible range of definitions of ‘youth’ used in pertinent literature). Alternatively, at least 50% of a study’s sample had to be within that age range. We excluded studies of youths with chronic physical health conditions or a primary diagnosis of intellectual disability. Full texts were obtained for all potentially relevant studies. Two reviewers independently screened the full text of each article to check whether it met inclusion criteria.

The authors of six studies were contacted for additional information to determine their eligibility. Of these, three authors responded and provided data that had not appeared in the original studies, which were then included in our review.

### Data extraction

A data extraction sheet was created and refined following pilot testing on ten randomly selected included studies. Two reviewers independently extracted and compared data from all included studies and resolved disagreements by discussion.

We extracted data on participant demographics, study design, instruments used, study setting, healthcare context, pathways to care, and measures of treatment delay. If needed, authors were contacted for clarifications or missing information.

The two reviewers also independently ascertained the quality of each included study using a rating scale adapted from the Newcastle–Ottawa Quality Assessment tool [20], which had been used in a systematic review on pathways to care in first-episode psychosis [21] (see online supplementary material).

## Results

**Fig. 1:**
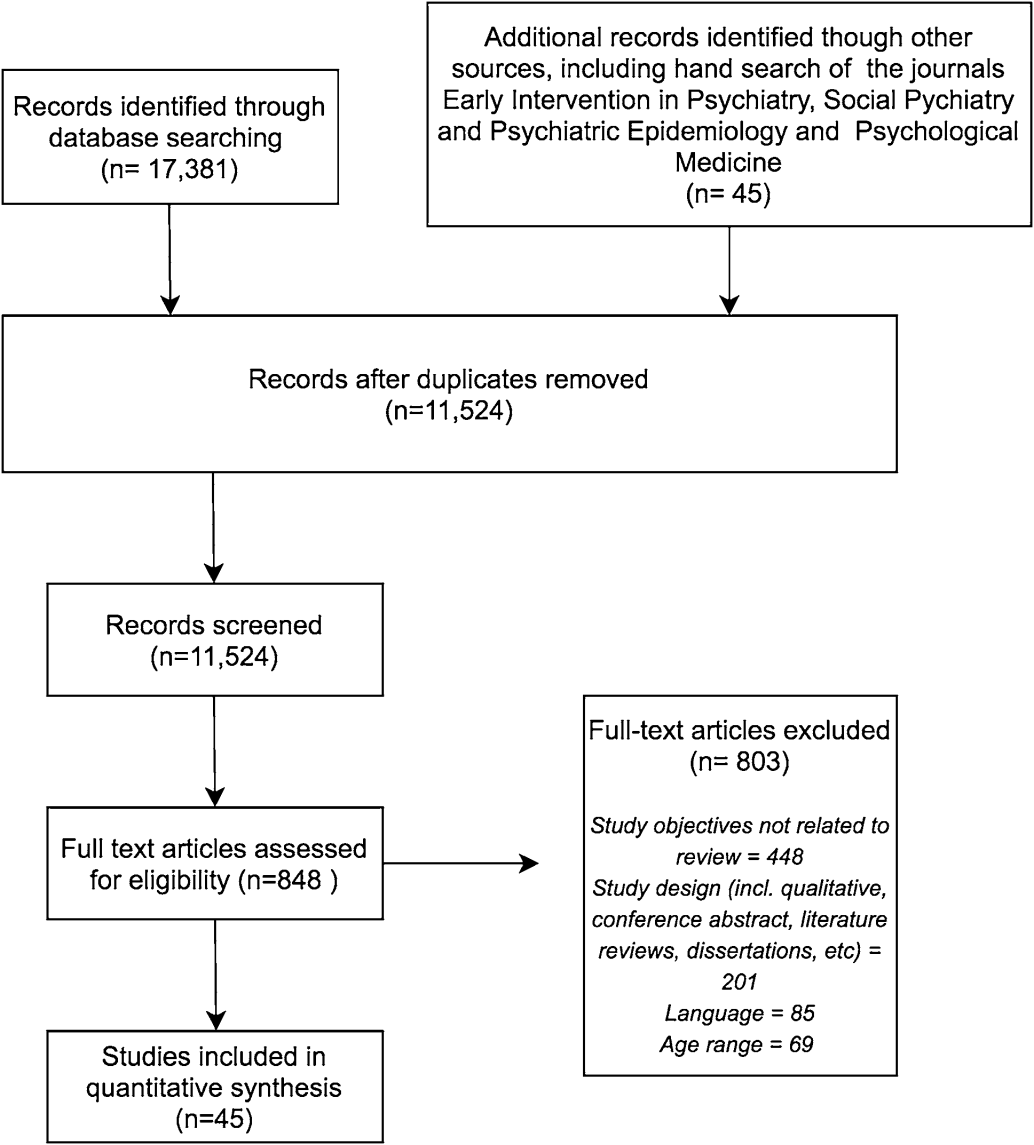
PRISMA flow chart of included studies

The electronic search yielded 17,381 publications, including 1454 from the March 2018 search update. Hand searching yielded another 45 articles. After duplicates were removed, 11,524 studies remained. Initial title and abstract screening identified 845 potentially relevant studies for full-text screening. Of these, 45 studies fulfilled the inclusion criteria (see Fig. 1). The main reasons for exclusion were misalignment of studies’ objectives with those of this review, study methodology, language, and participants’ age ranges. Five studies were excluded post hoc because their participants’ age ranges could not be established (*n* = 3), or for involving the same participants as other included publications (*n* = 2).

### Study characteristics and settings

The characteristics of included studies are summarized in Table 1. The studies were conducted across a wide range of countries (*n* = 26). Their sample sizes ranged from 15 to 1266 (mean = 203). Twenty-six studies were conducted in services catering to persons with first-episode psychosis. Other study sites were general psychiatric inpatient (*n* = 3) and outpatient units (*n* = 9); and specialized services for youths with anorexia (*n* = 1) and those at risk for psychosis (*n* = 6).

**Table 1:** Study characteristics

Study	Study objectives	Country	Population	Setting	*N*	Mean age (range)	(% male)	Instrument	Source of data
Addington et al. [22]	To determine the number of attempts it took before patients with FEP received adequate help, the signs or symptoms that led them to seek help and the people from whom they attempted to seek help	Calgary, Canada	First-episode psychosis	Early intervention for psychosis program	86	24 (15–51)	66.3%	Interview developed for the study	II + FI
Anderson et al. [12]	To estimate the extent to which sociodemographic, clinical, and service-level factors were associated with negative pathways to care and referral delay	Montreal, Canada	First-episode psychosis	Early intervention for psychosis program	324	Median 22.6 (14–30)	69.8%	CORS	II + FI + CR
Anderson et al. [23]	To compare the pathways to care and duration of untreated psychosis for people of Black African, Black Caribbean, or White European origin with FEP	Toronto and Hamilton, Canada	First-episode psychosis	Early intervention for psychosis program	171	21 (19–27)	66.7%	WHO Encounter Form	II + FI + CR
Archie et al. [24]	To examine ethnic variations in the pathways to care for persons accessing early intervention services in Ontario	Ontario, Canada	First-episode psychosis	Early intervention for psychosis program	200	24.5 (16–50)	78%	CORS	II + FI + CR
Bakare [25]	To assess first points of contact and referral sources for a group of patients seen in a neuropsychiatric facility in South-Eastern Nigeria	Enugu, Nigeria	Any mental illness	Child and adolescent inpatient unit	393	15.7 (3–18)	55.7%	Interview developed for study	II + FI
Bekele et al. [26]	To describe the routes taken by patients to reach psychiatric care, evaluate the time delay before seeking psychiatric care, and investigate the relationship between delays in the pathway to care and sociodemographic and clinical factors	Addis Ababa, Ethiopia	Any mental illness	Mental health hospital (inpatient and outpatient)	1044	29 (2–85)	62.2%	WHO Encounter Form	II + CR
Bhui et al. [27]	To assess (1) which services or agencies are encountered by patients in their pathways to specialist psychiatric care; (2) which services or agencies and individual characteristics of patients were independently associated with the shortest DUP	East London, UK	First-episode psychosis	Specialist psychiatric service	480	67.7% under 30, (18–64)	61.3%	WHO Encounter Form	II
Chadda et al. [28]	To study the help-seeking behaviour of patients visiting a mental hospital	Delhi, India	Any mental illness	Outpatient clinic	78	50%+ under 30, (18–49)	61.5%	Questionnaire developed for study	II + FI + CR
Chesney et al. [29]	To describe the pathways to care for patients with FEP in Singapore	Singapore	First-episode psychosis	Early intervention for psychosis program	900	27.1, (16–40)	49.7%	Interview developed for study	II + CR
Cheung et al. [30]	To estimate the public health costs of specific help-seeking pathways into an early intervention psychosis clinic	Edmonton, Canada	First-episode psychosis	Early intervention for psychosis program	50	22.2	82.0%	Semi-structured interview (PCI)	II
Chiang et al. [31]	To review the help-seeking pathways and reasons for delay for patients with FEP	Hong Kong	First-episode psychosis	Early intervention for psychosis program	55	22.2 (16–30)	60.0%	Interview developed for study	II + FI
Chien and Compton [32]	To explore the possible effects of mode of onset on pathways to care	Atlanta, United States	First-episode psychosis	Hospital for FEP psychiatric units	76	Mean 23.2	77.6%	Interview developed for study	II
Commander et al. [33]	To compare the experiences of people with non-affective psychoses from three broad ethnic groups, with respect to (a) pathways to care (b) the treatment received while in hospital (c) the delivery of care post-discharge	Birmingham, UK	First-episode psychosis	4 hospital inpatient units	120	65% under 35 (16–60)	59.1%	WHO Encounter Form	II
Compton et al. [34]	To examine the pathways to care and number of help-seeking contacts prior to hospitalization in first-episode patients of African–American background, and to ascertain the frequency of contact with primary care providers and police	Atlanta, United States	First-episode psychosis	Public sector hospital or crisis centre (inpatient)	25	22.8 (18–32)	76.0%	Symptom onset in schizophrenia inventory, CORS	II
Cougnard et al. [35]	To describe the pathways to care between onset of psychosis and first admission	Bordeaux, France	First-episode psychosis	Acute wards of two psychiatric hospitals	85	27.8 (17–45)	63.9%	Questionnaire developed for study	II + FI + CR
Del Vecchio et al. [36]	To explore the role of relatives in pathways to care of patients with a recent onset of psychosis	Naples, Italy	First-episode psychosis	Outpatient unit	34	26 (18–35)	64.7%	Pathways to care Form	II
Ehmann et al. [37]	To examine the treatment delay associated with community and inpatient pathways into care for persons experiencing FEP	Vancouver, Canada	First-episode psychosis	Early intervention for psychosis service	104	20.9 (15–37)	67.3%	WHO Encounter Form	II + FI
Etheridge et al. [38]	To assess whether duration of untreated psychosis in Rotherham reflected that reported nationally and internationally, and to identify potential obstacles to early identification and treatment	Rotherham, UK	First-episode psychosis	Early intervention for psychosis services (inpatient and outpatient)	18	29.4 (15–50)	61.1%	Questionnaire developed for study	II + FI
Fridgen et al. [39]	To examine the help-seeking behaviour of individuals at risk for psychosis or with FEP in a low-threshold system with easy access to mental health care facilities, in which a specialized early detection clinic was newly established	Basel, Switzerland	First-episode psychosis	Early intervention for psychosis outpatient clinic	61 UHR + 37 FEP	28.4 (18+)	59.0%	Basel interview for psychosis	II
Fuchs and Steinert [40]	To examine patients’ help-seeking contacts and the delays on their pathways to psychiatric care in Germany	Ravensburg, Germany	First-episode psychosis	Admission in hospital for first-episode psychosis	66	Median 26 (14–51)	59.0%	IRAOS + interview, adapted	II
Giasuddin et al. [41]	To find out the referral patterns, delays to reach mental health professionals, and diagnoses and treatment received before reaching psychiatric care	Dhaka, Bangladesh	Any mental illness	Outpatient clinic	50	25.8 (12–45)	58.0%	WHO Encounter Form	II
Hastrup et al. [42]	To document DUPs in Denmark and investigate associations of DUP with demographic characteristics, premorbid and illness-related factors and health-service factors	Denmark	First-episode psychosis	General population with FEP diagnosis	1266	21 (15–25)	55.5%	Danish Psychiatric Register	CR
Hodgekins et al. [43]	To examine care pathways experienced by young people accessing a pilot specialist youth mental health service for those with non-psychotic, severe, and complex mental health conditions	Norfolk, UK	Any mental illness	Specialist mental health service	94	18.3 (14–25)	28.7%	Interview developed for study	II or FI + CR
Jain et al. [44]	To evaluate the pathway to care of mentally ill patients attending a tertiary mental health facility in Jaipur, to highlight the difficulties of the mentally ill and their relatives in accessing appropriate care	Jaipur, India	Any mental illness	Tertiary mental health facility	76	59% under 30	71.5%	WHO Encounter Form	II + FI
Judge et al. [45]	To examine the duration of untreated psychosis in an FEP population, to describe precipitants of help-seeking attempts, and to identify barriers to obtaining appropriate treatment	North Carolina, USA	First-episode psychosis	Early intervention for psychosis clinic	20	19.8	75.0%	Pathways to care interview (Perkins)	II
Kurihara et al. [46]	To trace the help-seeking pathway of mental patients and to elucidate the role of traditional healing	Bali, Indonesia	Any mental illness	Admission to Mental Hospital	54	30.6	48.0%	Interview developed for study	II + FI + CR
Lahariya et al. [47]	To study the sociodemographic profile of psychiatric patients; to understand the pathways to care of the patients attending the facility, and to explore the interrelationships between pathways to care and sociodemographic variables	Gwalior, India	Any mental illness	Outpatient department of a psychiatric hospital	295	16–45	68.8%	WHO Encounter Form + interview	II
Lincoln et al. [48]	To gain an understanding of treatment delays in light of an initial episode of psychosis through examination of pathways to care	Melbourne, Australia	First-episode psychosis	Early intervention for psychosis program	62	22.8 (16–30)	64.5%	WHO Encounter Form	II
McMiller and Weisz [49]	To determine whether African–American and Latino families were less likely than Caucasian families to seek help from agencies and professionals prior to contacting clinics for their child	California, USA	Any mental illness	Community mental health clinic	192	11.4 (7–17)	64.0%	Referral sequence and problems interview	II + FI
Mkize and Uys [50]	To determine the pathways of care that clients with mental illness take, the effects of socio-cultural and economic factors on the pathways to mental health care and the satisfaction with different service providers consulted	Natal, South Africa	Any mental illness	Admission to a mental health institution	15	67% below 29 (15–59)	46.7%	Interview developed for study	II
Naqvi et al. [51]	To systematically study the care and referral pathways taken by patients before they present to a psychiatrist at a university teaching hospital	Karachi, Pakistan	Any mental illness	Outpatient psychiatry clinic	94	53% under age 30	55.3%	Interview developed for the study	II
Neubauer et al. [52]	To investigate the duration of untreated illness and paths to first treatment in early vs intermediate vs late age of onset anorexia nervosa	Varied institutions, Germany	Anorexia	Specialized services for anorexia (inpatient and outpatient)	140	22.3	All female	Multiple choice questionnaire developed for study	II
Norman et al. [53]	To examine and compare the extent of delay in individuals contacting health professionals and the delay in receiving treatment once such contact is made	London, Canada	First-episode psychosis	Early intervention for psychosis program	110	26.2 (16–51)	80.0%	CORS	II + CR + FI
O’Callaghan et al. [54]	To establish if, when and where people seek help in the early phase of psychosis in a representative sample	Dublin, Ireland	First-episode psychosis	Community-based psychiatric services	142	30.5 (16–64)	62.0%	Beiser scale for DUP; interview for pathways	II
Phillips et al. [55]	To summarize patterns of referral to one service providing clinical care for young people known to be at high risk of developing a psychotic illness	Melbourne, Australia	Ultra-high risk for psychosis	Specialized clinical service	162	18.8 (14–30)	61.0%	Interview developed for study	II + FI
Platz et al. [56]	To obtain information about type of health professionals contacted by patients on their help-seeking pathways; number of contacts; type of symptoms leading to contacts; interval between initial contact and referral to a specialized service	Switzerland	First-episode psychosis, ultra-high risk for psychosis, help-seeking but not UHR or FEP	Specialized outpatient service for UHR	104	22 (14–40)	73.0%	Interview developed for the study	II
Reeler [57]	To investigate pathways to care	Harare, Zimbabwe	Any mental illness	Psychiatric inpatient unit	48	28.2	31.1%	WHO Encounter Form	II
Reynolds et al. [58]	To explore the impact of a general practitioner training programme on referrals and pathways to care for people at high clinical risk of psychosis or with a first-episode psychosis	Southwark, UK	First-episode psychosis	Early intervention for psychosis program	102	21.9(UHR) 24 (FEP)	59%, (UHR), 75% (FEP)	Chart review methodology	CR
Sharifi et al. [59]	To conduct a first study on the duration of untreated psychosis and pathways to care among patients with first-episode psychosis in Iran as a developing country	Tehran, Iran	First-episode psychosis	Admission to psychiatric hospital	91	27.4	58.2%	Interview developed for the study	II + FRI + CR
Shin et al. [60]	To examine patients’ help-seeking contacts in a context (Korea) where pathways to care had not been examined before	South Korea	Ultra-high risk for psychosis	Early intervention for psychosis programs	18	15.8 (15–18)	72.2%	Interview developed for the study	II + FI
Stowkowy et al. [61]	To prospectively investigate the pathways to care of those at clinical high risk of developing psychosis	Toronto, Canada	Ultra-high risk for psychosis	Clinic for ultra-high risk of psychosis	35	21 (14–30)	71.4%	Pathways to care interview (Perkins)	II + FI
Subramaniam et al. [62]	To create a typology of patients with first-episode psychosis based on sociodemographic and clinical characteristics, service use and outcomes using cluster analysis	Singapore	First-episode psychosis	Early intervention for psychosis program	900	27.1 (15–41)	49.6%	Chart review	CR
Turner et al. [63]	To present the clinical and sociodemographic characteristics of patients referred to an early intervention for psychosis service and to describe their pathways to care	Christchurch, New Zealand	First-episode psychosis	Early intervention for psychosis program	182	22.4 (16–30)	72.5%	Interview developed for the study	II
Graf von Reventlow et al. [64]	To acquire accurate knowledge about pathways to care and delay in obtaining specialized high risk care	Finland, Germany, Netherlands, UK	Ultra-high risk for psychosis	Early intervention for psychosis program	233	23	54.9%	WHO Encounter Form, EPOS Form	II
Wiltink et al. [65]	To investigate if the drop in rates of transition from ultra-high risk to FEP may be due to potential changes in patterns of referral to a large ultra-high risk clinic	Melbourne, Australia	Ultra-high risk for psychosis	Early intervention for psychosis program	150	18.3	44.0%	Interview developed for the study	II + CR

DUP, duration of untreated psychosis; CORS, Circumstance of Onset and Relapse Schedule; CR, chart review; FEP, first-episode psychosis; FI, family interviews; II, individual interviews; IRAOS, Instrument for the Retrospective Assessment of the Onset of Schizophrenia; PCI, Pathways to Care Interview; UHR, ultra-high risk

### Healthcare system and organizational contexts

We extracted information about the healthcare system in which each study was conducted (Table 2). Many studies described organizational features, including available healthcare tiers (e.g., public/private) and local practices (e.g., preference for traditional healers). Fourteen studies reported allowing open referrals, wherein direct referrals to the services were possible. Two studies described a gatekeeper system where referrals from primary care were required to access mental healthcare. All other studies did not specify their settings’ referral systems.

**Table 2:** Study outcomes I—Pathways to care, treatment delays and health system contexts across studies

Study	Pathway to care definition	Pathway to care timeframe	Pathways to care (number of help-seeking contacts)	Treatment delays, in weeks	Notes on health system context
Addington et al. [22]	The number of individuals who were sought out for assistance with mental health concerns	From onset of psychosis to EI service	Pre-onset: mean 1.7, range 1–4 After onset: mean 2.3, range 1–6	DUP mean 102, median 27, range 0–780	Comprehensive program for individuals experiencing their first episode of psychosis. It is predicted that 80–90% of all new cases in Calgary are being referred to this specialized program
Anderson et al. [12]	Type and sequence of contacts that the patient or family member sought help from	Lifetime until entry to EI service	Median 3	DUI median 194.4, DUP median 16.4 Referral delay median 1	Only specialized service for treatment of FEP within catchment area. Patients referred from any source
Anderson et al. [23]	Series of help-seeking contacts made by patients and their family members in response to the symptoms of a mental illness	Onset of psychotic symptoms to contact with EI service	Median 6 (White Europeans); Median 4 (Black African and Black Caribbean)	Black Caribbean DUP median 69.5, White European DUP median 30.4, Black African DUP median 39.1	Hospital and community-based early intervention services for FEP in two cities
Archie et al. [24]	Sequence of all formal and informal supports contacted by participants seeking help	Onset of psychosis—entry to service	Mean 2.9 (SD = 2), median 3	DUP mean 60.6, median 22.1, SD 11.2	Specialized services within catchment area Referrals accepted from all sources (including self-referrals)
Bakare [25]	Places where help was sought	Prior to presenting to hospital	NS	NS	Healthcare system is divided between primary, secondary, and tertiary care. Patients are free to access any tier of healthcare without referral
Bekele et al. [26]	The routes taken by patients to reach psychiatric care	NS—(WHO Encounter Form uses previous 12-month timeframe)^a^	Range 0–4 contacts	Median 38, range: less than 1–45 years	Only mental hospital that provides outpatient and inpatient services for the full range of psychiatric disorders in the entire country. Patients can refer themselves directly to services
Bhui et al. [27]	The services/agencies encountered by patients in their pathways to specialist psychiatric care	NS—(WHO Encounter Form uses previous 12-month timeframe)^a^	Range 0–3. 13% were in contact with psychiatric services at first contact; 73.33% at second contact, and 97.71% at third contact	Median 12, IQR 1–9.5	The East London First Episode Psychosis Study was a large, population-based incidence study in three neighbouring boroughs
Chadda et al. [28]	The various treatment services utilized by a group of psychiatric patients visiting a mental hospital	From onset of illness to mental health hospital	Range 0–3	Median 78. Help-seeking median 52, range 4 days–20 years	Catchment area serving 30–40 million population. Facilities for psychiatric treatment are generally available in general hospital psychiatric units, mental hospitals and office-based practice. In India, mental hospitals remain one of the major service providers to the mentally ill
Chesney et al. [29]	The individuals and organizations who are contacted by patients and their carers in order to seek help and receive treatment	Sources of help until referral to EI service	Mean 2.7 (SD, 0.9), median 3, range, 1–7	Mean 53.6, median 20, range 0–204, SD 24.3	The only state mental hospital in Singapore, single largest tertiary care facility in Singapore
Cheung et al. [30]	Sequence of contacts with individuals and organizations in seeking help	Post-onset and up to 1 year prior to admission/intake at the early psychosis clinic	Mean 4.48 (inpatient pathways), mean 2.68 (outpatient pathways)	NS	Specialized FEP clinic within a public health service responsible for a region of approx. 1 million people
Chiang et al. [31]	Help-seeking contacts before treatment in the EASY programme, a service for early psychosis	NS	Mean 1.06	DUP mean 23.5 for GP first contact; mean 60 for private psychiatrist; mean 36.2 for helpline; mean 1.49 for ER	The programme accepts referrals of patients with FEP aged between 15 and 25 years, with an open referral system
Chien and Compton [32]	The various help-seeking contacts made between the onset of illness and engagement in treatment	Onset of illness to engagement in treatment	Mean 2.2 (SD 1.5), range 1–8	Mean 27.7	Urban, public sector psychiatric units
Commander et al. [33]	Past history of involvement with forensic and psychiatric services	48 h prior to admission	30% of Asian group, 45% of Black group, 10% of White, and 10% of White group had over 3 contacts	NS	Four hospitals providing most inpatient care in Birmingham
Compton et al. [34]	Any help-seeking attempt initiated for the purpose of evaluating or treating either prodromal or psychotic symptoms	From the onset of prodromal symptoms until first hospital admission	Mean 3.3 (SD 2.0), range 1–8	DUI mean 146.4, median 128, SD 151.3, range: 0.6–476.9. DUP mean 65.3, median 32.9, SD 89.1, range 0.4–337.7. Help-seeking delay mean 88.6 median 48.7, SD 48.7, range: 0.6–394.9	Public sector outpatient services are available, though this sample focused on patients requiring hospital admission
Cougnard et al. [35]	Number and profession of successive helping contacts, and the treatment and referral proposed by each contact	Between onset of psychosis and first admission	Median 2, range 1–7	Help-seeking delay median 9. Median delay to first treatment 28. Median delay to admission 52	Universal access to care with free access to private or public mental health professionals
Del Vecchio et al. [36]	Pathways to psychiatric care	NS	Mean 0.8 (SD 0.8)	DUP mean 33.3 SD 54, DUI mean 145.4 SD 141.9. Help-seeking delay mean 17.6 SD 45. Referral delay mean 15.6 SD 29.9	NS
Ehmann et al. [37]	Help-seeking efforts leading up to referral to program’	Onset of psychosis to referral to program	Mean 3.02 (SD 1.31), range 1–7	Mean 92, median 30.5, SD 131, range 1–691	Single EI program for psychosis within a defined catchment area; accepts referrals from any source
Etheridge et al. [38]	Experiences of obtaining care when they first developed symptoms of psychosis	From when the illness started to referral	NS (service users), mean 3 (carers, on behalf of service users)	67% had DUI less than 52, 22% between 52 and 156, 11% more than 1	Swallownest Court Services, including the rehabilitation ward, assertive outreach service and day hospital
Fridgen et al. [39]	Person contacted first along the help-seeking pathway and which persons or institutions were contacted subsequently	Any help-seeking attempt before coming to the early detection clinic	Mean 1.5, median 1, range 0–6	DUI median 177, DUP median 52. Referral delay mean 165, median 39	Psychiatrists in private practice and general practitioners, both with the possibility of referring to the university outpatient clinic
Fuchs and Steinert [40]	Professional contacts	Before admission	42% had more than 1 contact, range 1–5	Mean 71; median 8 Help-seeking delay mean 5	Sole psychiatric hospital in catchment area. Patients can consult outpatient psychiatric care without a referral
Giasuddin et al. [41]	Initial and intermediate carers, and number of steps needed to reach mental health personnel	From symptom onset to arrival at a psychiatric service	Mean 2.7	DUI mean 48, Median 25; Range 1–156. Help-seeking mean 13.8	Direct access to specialized care is permitted
Hastrup et al. [42]	Referral source was defined as general practitioner, emergency wards or other hospital services Contact leading to FEP diagnosis was reported as either with an inpatient or an outpatient unit	Interval from onset of psychotic symptoms to initiation of appropriate treatment (antipsychotic medication)	NS	32.7% had a DUP below 26, 17.7% had DUP between 26 and 52. 32.8% had a DUP longer than 52	Danish National Indicator Project (DNIP). In Denmark, it is mandatory for all psychiatric hospital units and relevant clinical departments to report data on all patients with schizophrenia to the registry
Hodgekins et al. [43]	Sequence of help-seeking contacts with individuals and organizations	From date of onset	Mean 5.53	Mean delay 195; Mean help-seeking delay 70.9; Mean referral delay 118.4	Pilot specialist youth mental health service for young people aged 14 to 25 years with non-psychotic, severe and complex mental health conditions
Jain et al. [44]	Sources of care used by patients before seeking help from mental health professionals and also the factors that modify it	From onset to visit with mental health professionals and to tertiary care centre	Total mean 5.3 (SD 10.7), median 2, range 0–67 Mean before reaching any mental health professional: 3.9 (SD 6.7), median 2, range 1–51	Mean DUI 212, Median 56, Range 1–1042	Patients allowed to seek help from any source of their choice and this includes faith healers. Government-run tertiary care centre providing free treatment to catchment area
Judge et al. [45]	Each help-seeking attempt to whom participants turned for help	Onset of psychosis and administration of antipsychotic medication^a^	Mean 5.1, range 1–15	DUP mean 83.4, range 8–312 From onset to recognition = 33.8, from recognition to treatment = 63	The only specialized psychotic disorders clinic in a catchment area, which ranges from suburban to rural
Kurihara et al. [46]	All sources of care sought	Prior to visiting mental hospital	NS	DUI to hospital admission median 26 Help-seeking delay median 6 Referral delay to hospital median 12	Access to both general practitioners and community health centres is readily available. In Bali, mental disorders are commonly considered ‘non- medical diseases’ thought to be the domain not of doctors, but of traditional healers
Lahariya et al. [47]	A pathway a patient adopts to reach the appropriate treatment centre	NS (WHO Encounter Form uses previous 12-month timeframe)^a^	NS	DUI 45.6	Outpatient department of a specialty psychiatric hospital affiliated with medical college in the city
Lincoln et al. [48]	Range of people to whom individuals turn to for help	NS (WHO Encounter Form uses previous 12-month timeframe)^a^	Mean 4.9 SD 2.8, median 4.5, range 1–17	DUP mean 38.8, median 17.2. Help-seeking delay mean 16, median 4.4	Comprehensive and integrated community-based service for young people with FEP
McMiller and Weisz [49]	Sequence of consultations and referrals preceding child clinic intake	Prior to contact with mental health clinic	NS	NS	NS
Mkize and Uys [50]	Actions taken by individuals towards the early detection of mental illness. Specifically, steps or consultations taken by the client before being admitted to a mental health institution	Time of the onset of mental illness to the time of their admission to a mental health institution	NS	Range 26–130	NS
Naqvi et al. [51]	Care and referral pathway before presenting to a psychiatrist, including all professional and non-professional avenues	Since the onset of symptoms to appropriate care	Median 2	Help-seeking delay mean 146, range 1–6 years Delay from first contact to psychiatrist mean 198	Most mental health facilities are in urban areas, but are under-resourced. No referral system in operation
Neubauer et al. [52]	Previous treatment facilities and paths to first treatment	Between onset and initiation of treatment	NS	Mean DUI = 109, SD, 160, range 0–843	German healthcare system, details not specified
Norman et al. [53]	All formal services, organizations or professional services consulted regarding any mental health/psychiatric problems experienced by the patient	Lifetime until entry to EI service	NS	Mean DUP 61.1, median 21, SD 100.8. Help-seeking delay mean 25.1, SD 58.5. Referral delay mean 44.6, SD 88.5	EI service with open referral system within a public healthcare system^a^
O’Callaghan et al. [54]	All previous contacts with health services, the police and the judiciary, and any treatment received	From 28 days prior to onset of prodrome to entry to EI service	Median 2, range 0–8	Mean DUP 82; DUI 180. Delays evenly split between help-seeking and referral delays	Catchment area-based psychiatric services receiving referrals from general practitioners and emergency departments
Phllips et al. [55]	Previous contacts made with health and allied services	Prior to referral	Mean 2.36, SD 1.32, range 1–7	Total delay mean 127. Help-seeking delay mean 85.8, SD 132.71. First contact to treatment delay mean 41.4, SD 91.4	Specialized clinical/research service for young people thought to be at high risk of developing a psychotic episode
Platz et al. [56]	Professional groups that individuals had previously contacted for similar problems	Previous contacts	Mean 2.38, SD 1.4, median 3, range 1–8; no difference between UHR, FEP and help-seeking others	First contact to referral for UHR: mean 124, median 36, SD 217.1, range 1 day–7.6 years Referral delay median for UHR, FEP and help-seeking others = 28 Median help-seeking delay lower for FEP than for UHR and help-seeking others	Semi-urban catchment area of part of the only general psychiatric outpatient clinic. Patients can refer themselves directly to any public or private psychiatric facility and do not require referrals
Reeler [57]	Various carers, kinds of treatment offered, and the times of various events	NS (WHO Encounter form 12 months)	NS	Help-seeking delay range 1–56.4; referral delay range 4.4–50.5	Filter model of service, with stress on a primary care base
Reynolds et al. [58]	Referrals and pathways to care to specialized early intervention service following trainings to general practitioners	NS	Range 1–5	NS	Community-based team accepts referrals from any source
Sharifi et al. [59]	Pathways that patients take to reach psychiatric care (admission to psychiatric hospital)	Any previous helping contacts and referrals	NS	Mean 52.3, median 11	Care to patients with mental illnesses is delivered by public and private sectors. Patients and their families select their own care provider
Shin et al. [60]	The contact process from when the illness is suspected until the first psychiatric treatment	From the initial suspected psychiatric illness until the first psychiatric help was noted	Median 0.7, range 0–4	Mean 53.24, SD 50.28 DUI mean 56.49, range: 2 –156	The Korean public health system does not provide a GP and therefore seeking psychiatric help is initiated by patients themselves. Each centre is main provider of psychiatric services in their area
Stowkowy et al. [61]	All help-seeking activities collected in chronological order from onset of prodromal symptoms	For the period from the onset of prodromal symptoms to referral to clinic	Mean 1.7, range 1–4	NS	UHR clinic accepting referrals from all sources
Subramaniam et al. [62]	The sources of help sought in chronological order till the patients were referred	First contact to admission	Mean 3.2, range 1–7	DUI mean 26, DUP mean 21.7	Comprehensive, integrated, multidisciplinary and patient-centred program
Turner et al. [63]	Patients’ contact with social agencies prior to entering EI service	6 months prior	Mean 3.87 (SD 6.31), range 0–42	DUP mean 17.14 for schizophrenia; DUP mean 4.14 for affective and other psychosis	The service available to all those with first-episode psychosis referred into the only early intervention for psychosis service in the Christchurch catchment area
Graf von Reventlow et al. [64]	Number of help-seeking events from onset of at-risk criteria to receiving appropriate treatment	The period between the onset of frank psychosis and receiving an adequate treatment	Mean 2.9	DUI mean 182.5, help-seeking delay mean 72.6. Referral delay mean 110.9	Public sector mental health care (Finland, the UK) and private mental healthcare sector providing beds in psychiatric hospitals (Germany, the Netherlands)
Wiltink et al. [65]	When a (health) service was first contacted, how many and which other services were contacted after that, and who made the referral	From onset to referral to clinic	Mean 1.93	Total delay 46.5. Referral delay 6.5	The catchment area-based program with open referral system

DUP, duration of untreated psychosis; EI, early intervention; ER, emergency room; FEP, first-episode psychosis; IQR, inter-quartile range; NS, not specified; SD, standard deviation; UHR, ultra-high risk

^a^Inferred from text, not explicitly stated

### Instruments and data sources

Studies differed in the instruments used to ascertain pathways to care. The majority had developed their own interview guide or questionnaire (*n* = 22) but provided limited to no information on the methodology used to develop the measures or their psychometrics. Semi-structured interview-based instruments included the WHO Encounter Form [9] (*n* = 14); the Circumstances of Onset and Relapse Schedule [66] for early psychosis (*n* = 4); the Pathways to Care Schedule [67] (*n* = 3); and the Basel Screening Instrument for Psychosis [68] (*n* = 1). One study used the structured Referral Sequence and Problem Interview [49].

Irrespective of the instruments used, most studies collected and corroborated information from multiple sources (*n* = 27). In these cases, individual interviews were supplemented by family/carer interviews and/or chart review. Some studies relied on a single data source—patient interviews (*n* = 16) or chart information (*n* = 2). One study used national registry data, which included healthcare contacts and durations of untreated illness.

### Timeframes

Timeframes for delimiting pathways to care, i.e., the start and endpoints of journey into care, differed widely across studies. Startpoints included the onset of symptoms or initial suspected illness (*n* = 22); 6 months preceding entry (*n* = 1); lifetime (*n* = 4); 28 days preceding prodromal symptom onset (*n* = 1); 48 h prior to admission (*n* = 1); and first contact with health services (*n* = 1).

Endpoints included entry or referral to a specialized service (*n* = 13); admission to hospital (*n* = 8); initiation of care (*n* = 7); and entry to a general psychiatric service (*n* = 5). For studies that did not specify a timeframe but used the WHO Encounter Form (*n* = 5), we assumed that instrument’s stated timeframe of 12 months preceding the interview (see Table 2). Other studies did not specify clear start (*n* = 10) or endpoints (*n* = 7).

### Pathways to care

The focus of this review was on articles that examined individuals’ pathways to care (i.e., sequence or number of help-seeking contacts). Outcome measures included descriptions of full trajectories, or first and last contacts before a specific endpoint. Considered clinically relevant, first and last contacts are often described in pathways to care studies [16].

Thirty-five studies described full pathways to care sequences, including the total number and types of contacts in individual participants’ pathways to care. Seven studies described the most common pathway contacts for their sample, in addition to common first and last contacts. Three studies described the most common overall and first contacts along participants’ pathways to care (see Table 3).

**Table 3:** Study outcomes II—Help-seeking contacts across studies

Authors	Key pathway agents	Common first help-seeking contacts	Common referral sources
Addington et al. [22]	Most common: emergency services (33%), family physicians (23%) Other: psychologists, teachers/counsellors, psychiatrists, family, emergency services, police, clergy, social workers, and friends		Emergency services (52%), family physicians (18%), psychiatrists (18%)
Anderson et al. [12]	Over 45% of patients had contact with police or ambulance	Emergency services (62%)	Emergency services (74%)
Anderson et al. [23]	Primary care physicians are most commonly used overall	Most common: primary care physicians	Most common: inpatient units
Archie et al. [24]	Most common: emergency services and primary care physicians, family, doctors/walk-in clinics, clergy/homeopath/other non-medical contacts, psychologists, psychiatrists, school counsellors, psychiatric admissions	Family doctor/walk-in clinic (31%), emergency services (24%), clergy/homeopath (12%)	Psychiatric admissions (40.2%), family doctor/walk-in clinic (14.8%), emergency services (13.8%)
Bakare [25]	Neuropsychiatric hospitals, prayer houses, other hospitals, traditional healers, patent medicine stores, roadside medical labs, specialized school for children	Psychiatric hospitals (48%), prayer houses (22%), other hospitals (21%)	Relatives, family, or friends. (92%), other hospitals (7%), prayer houses/faith healing centres (1%)
Bekele et al. [26]	Priests, herbalists, nurses, doctors	Priests/holy water (31%), doctors (21.5%), herbalists (4.5%)	Self-referrals (41%)
Bhui et al. [27]	Primary care physicians, emergency services, police, community-based health and social care agencies, prisons, psychiatric services, native or religious healers	Primary care physicians, emergency services, and criminal justice agencies	
Chadda et al. [28]	Traditional healers, psychiatrists, non-psychiatric doctors, Ayurveda (Indian system of herbal medicine)	Psychiatrists (58%), religious faith healers (30%), physicians (12%)	
Chesney et al. [29]	Medical specialists, psychiatrists, private psychiatrists, direct referrals, at-risk clinic, primary care physicians, health professionals, counsellors community health assessment team, police, employers and teachers, other, traditional or religious healers, courts, lawyers	Specialist care (59%), primary care (27%), police (12%)	Thirty patients (3%) were self-referred
Cheung et al. [30]	Teachers, counsellors, police, psychologists, psychiatrists, family physicians, emergency services, public health, outpatient psychiatry, other		
Chiang et al. [31]	Self-referral, medical, non-medical and religious, alternative help	Social workers, primary care physicians	Telephone helpline, emergency services, primary care
Chien and Compton [32]	Hospital/emergency services, police, outpatient service, family physicians	Psychiatric hospital and emergency (32%), psychiatrists, counsellors, or outpatient mental health clinics (26%), police (20%)	Psychiatric hospitals, psychiatric or general emergency services, police (25%), psychiatrists, counsellors, or outpatient mental health clinics (13.2%), emergency services (7.4%)
Commander et al. [33]	Psychiatrists, social workers, police, emergency services, primary care physicians, community psychiatric nurses, other, self		
Compton et al. [34]	Most common: mental health professionals and psychiatric emergency services, general emergency department, primary care physicians, police, other	Mental health professionals (32%), psychiatric emergency services (24%), general emergency departments (20%)	Psychiatric emergency services (36%), mental health professionals (20%), general emergency departments (20%), police (20%)
Cougnard et al. [35]	Primary care physicians (32%)	Primary care physicians (37%), psychiatrists	
Del Vecchio et al. [36]	Primary care physicians, psychiatrists, neurologists, psychologists, relatives	Primary care physicians (28%), psychiatrists (30%), neurologists (21%)	
Ehmann et al. [37]	Relatives/friends, schools, counsellors or crisis line, mental health teams, general physicians, private psychiatrists, hospitals, direct entry		Relatives/friends (52%), primary care physicians (16%), self-referrals (9%), counsellor or crisis line (8%), mental health teams (6%), psychologists (5%)
Etheridge et al. [38]	Primary care physicians, relatives, psychiatrists, teachers, hospitals	Most common by service users: relatives, primary care physicians, psychiatrists, teachers and hospitals Most common by family/carers on behalf of a service user: primary care physicians, school staff, police and emergency services	
Fridgen et al. [39]	Friends, family, psychiatrists, primary care physicians, colleagues, partners, other physicians, psychologists, priests, alternative medicine	Family or friends (46%), private psychiatrists (14%), or primary care physicians (12%)	Outpatient departments, private psychiatrists, other physicians, self-referrals, family
Fuchs and Steinert [40]	Most common: mental health professionals (46%), primary care physicians (20%), hospitals (18%), and psychosocial contacts (16%)	Primary care physicians (18%)	
Giasuddin et al. [41]	Private practitioners, native or religious healers, other medical facilities, general hospitals	Private practitioner (44%), native or religious healer (22%), direct pathway (16%)	
Hastrup et al. [42]	Primary care physicians, inpatient units, outpatient units, and emergency services, other medical specialists	Outpatient services (59%), hospital services (41%)	Emergency services (26%), primary care physicians (22%), hospitals (46%)
Hodgekins et al. [43]	Primary care physicians, education services, emergency services, social care, other	Primary care physicians, educational settings	
Jain et al. [44]	Faith healers, non-psychiatric allopath care providers, alternative medicine, direct entry, mental health professionals	Faith healers (40%), non-psychiatrist allopath care provider (29%), other psychiatrist (15%)	
Judge et al. [45]	Relatives, emergency services		
Kurihara et al. [46]	Most common: traditional healers. Others: primary care physcians, hospital doctors, community health centres	Traditional healers (43%), primary care physicians (7%), direct entry (4%)	Traditional healers (67%), community health centres (17%), and primary care physicians (13%)
Lahariya et al. [47]	Faith healers, psychiatrists, allopathic practitioners, traditional healers, other (friends and family)	Faith healers (69%), psychiatrists (9%)	Others (including previous patients), allopathic practitioners
Lincoln et al. [48]	Mental health professionals (50%), primary care physicians (17%)	Primary care physicians (36%), psychiatric services (16%), police (12%)	
McMiller and Weisz [49]	52% of all contacts were ‘professional’ (56% for Caucasians, 47% for African–Americans and 42% for Latino)	45% of first contacts were Healthcare professionals (53% for Caucasians, 32% African American, 30% Latino)	
Mkize and Uys [50]	Traditional healers, faith healers, hospitals, police, mental health institutions, primary health care clinics	Primary care physicians (33%), faith healers (20%), traditional healers (20%)	
Naqvi et al. [51]	Religious healers, primary care providers, specialists, hospitals doctors, psychiatric services		Self-referrals (49%), hospital or other specialists (20%), Primary care (2.9%)
Neubauer et al. [52]	Physicians, health professionals, mental health professionals, social networks, eating disorder clinics, day clinics	Inpatient treatment (55%), outpatient facility (39%), eating disorder-specific centre (4%)	
Norman et al. [53]	Primary care physicians, community or school counsellors, psychologists, social workers, psychiatrists, hospitals, emergency services	Before psychosis: primary care physicians (40%), community or school counsellors (30%), psychologists or social workers (20%) After psychosis, hospital or emergency services (43%), primary care physicians (39%), community (13%)	Emergency services (49%), private psychiatrists or non-emergency hospital (26%), primary care physicians (15%)
O’Callaghan et al. [54]	Primary care physicians, emergency services, counselling services, police, religious organizations, complementary and alternative medical services, and clinic website	Primary care physicians (59%), other, including emergency services (41%)	
Phillips et al. [55]	Primary care physicians, private psychiatrists/psychologists, outpatient services, inpatient services, other		Youth access team, generic and mental health services, school and university counsellors and youth housing and employment workers
Platz et al. [56]	In-patient services, primary care physicians, alternative medical practitioners, non-medical counselling services, non-specified professionals	Primary care physicians (34.6%)	General practitioners, private psychiatrists/psychologists, psychiatric outpatient services
Reeler [57]	Hospital doctors, traditional healers	Hospital doctors	Hospital doctors
Reynolds et al. [58]	Primary care physicians, community-based teams, out of area teams, emergency services, police, prison, child and adolescent mental health teams, specialized services	Primary care physician (43%), emergency services (24%), police (11%)	Post training, 46% were referred by primary care physicians
Sharifi et al. [59]	Psychiatrists, primary care physicians, other health professionals, traditional healers, other professional	Psychiatrist (25%), traditional healer (23%) or a primary care physician (18%)	Family (33%), health professionals (32%) and the legal system (17%)
Shin et al. [60]	Most common: internet and family members (57%) Other: patients, teachers, physicians, specialized clinic, shelters		
Stowkowy et al. [61]	Primary care physicians, mental health clinics, psychiatrists and other individuals		Primary care physicians (29%), psychiatrists, mental health clinics and social workers, (14% each), Self-referral (11%)
Subramaniam et al. [62]	Primary care physicians, polyclinics, other primary care, hospitals, traditional or religious healers, direct entry, counsellors, police, courts, family, relatives, friends, other	Family, primary care physicians	Family
Turner et al. [63]	Primary care physicians, school counsellors, religious ministers, psychiatric outpatient clinics, private psychiatrists, other, mental health services, other health services		Inpatient services (64%), emergency services (16%), general practitioners (7.7%)
Graf von Reventlow et al. [64]	Physicians, psychiatrists, psychologists, nurses, social workers, treatment teams, other counsellors, other healthcare professionals, other professionals		
Wiltink et al. [65]	Primary care physicians, teachers, counsellors, drug and alcohol services, accommodation services, youth health services, emergency services, public hospital, other		

### Overall pathways

Twenty-eight studies reported the number of contacts before receiving specific services, which ranged from 0 to 15 contacts per participant (with a pooled mean across studies of 2.9 contacts). One study [43] dichotomized pathways into ‘short’ (three or fewer services before referral) and ‘long’ (four or more services). Its authors noted that the number of contacts did not always indicate pathway complexity or length of delay. For example, a pathway with many contacts could reflect appropriate referrals as mental health problems progressed, whereas shorter pathways could reflect repeated contacts with specific services or concurrent use of different services before an appropriate referral.

### Key pathway agents

Contacts involved in young people’s pathways to mental healthcare were varied and included medical professionals (general practitioners, psychiatrists); non-medical professionals (psychologists, social workers, counsellors, school teachers, rural healthcare workers); informal sources of help (family, friends, employers, colleagues); healthcare institutions (emergency services, inpatient units, walk-in clinics); criminal or justice system (police, prisons, lawyers, courts); traditional or faith-based healers (prayer houses, priests, herbalists, clergy); and technology-enabled contacts (websites, helplines, crisis lines) (Table 3).

#### First contact

Twenty-nine studies reported the first contact along the pathway to care. In order of frequency, young people’s first help-seeking contacts were general practitioners (14/29); psychiatrists or specialized services (5/29); faith or traditional healers (4/29); ERs/inpatient units (3/29); family or friends (2/29) and social workers (1/29). General practitioners were among the top three most frequent first sources of help in 24 of 29 studies.

#### Referral sources

Studies of pathways to care often describe their referral source as the ‘successful contact’, i.e., the contact that resulted in an individual obtaining the service in question. This successful contact is also referred to in some studies as the “last” contact. Twenty-two studies examined referral sources. Of these, eight described the ER/inpatient unit as the most common ‘successful’ referral source. Self-referrals (i.e., referrals made by youths themselves, or by family/carers on their behalf) were the most frequent referral source in six studies. Other prominent referral sources included general practitioners, general hospitals, helplines, and outpatient units.

### Treatment delays

Of the 39 studies that measured treatment delay, 23 were from first-episode psychosis settings, and 16 were from other mental health services (see Table 2).

#### Duration of untreated psychosis (DUP)

DUP is defined as the time between the onset of symptoms and the start of appropriate care (operationalized as the commencement of antipsychotic medication or admission to services). Across the 23 studies that reported DUP, mean DUP ranged from 1.5 to 102 weeks and median DUP ranged from 8 to 70 weeks. Of these 23 studies, 10 also assessed ‘help-seeking delays’ (time between the onset of initial symptoms and contact with the first pathway agent) and ‘referral delays’ (time between contact with the first pathway agent and the commencement of treatment at the study setting). Of these, three studies found that help-seeking delays exceeded referral delays [12, 34, 36]; six studies found referral delays to be longer, [35, 39, 40, 45, 48, 53]; and one study [54] found an even split between both delay components. Notably, FEP patients referred to a service for those at risk for psychosis reported longer referral, than help-seeking delays [56].

#### Duration of untreated illness (DUI)

Fifteen studies from a range of mental health settings described the length of treatment delays to their services. Although definitions of DUI varied, most studies conceptualized it as the time between the onset of symptoms and the commencement of treatment at their setting. DUI estimates ranged from 1 week to 45 years (Table 2). Despite our inclusion criteria focusing on young people between the ages of 11 and 30, the upper end of the range for DUI is 45 years. This is because we also included studies in which at least 50% of the included sample was in the age group of interest. Unfortunately, some of these studies did not break down their delay indices by age group (see Table 1 for participant characteristics for each included study.) At the very least, this wide range for DUI is indicative that there are often extremely lengthy delays before the receipt of appropriate treatment. Eight studies divided DUI into help-seeking and referral components. Of these, three studies reported lengthier help-seeking delays [28, 55, 56] and five reported lengthier referral delays [41, 43, 46, 51, 64].

### Impact of pathways to care on treatment delays

Seven studies found that encountering specific pathway agents affected treatment delay. One study [29] found that initial contacts with counsellors or courts led to longer DUPs. Another [42] found that DUP was shorter following referrals from emergency services. DUPs were shorter if the first contact was with general practitioners [59] and when comparing general practitioners to private psychiatrists and psychologists [56]. However, another study [12] reported longer referral delays for persons with FEP following contact with primary care, albeit such contact resulted in fewer negative pathways to care (e.g., emergency or inpatient services). In settings other than psychosis services, contacts with traditional or faith healers [46] or private general practitioners/physicians [41] were notably associated with longer DUIs. Family involvement during help-seeking was associated with shorter help-seeking delays in one study [54].

### Factors influencing pathways to care

Often, families/friends played a substantial role in the initiation of treatment. In two studies [26, 41], 70% of participants had sought mental healthcare on the advice of family. One of these studies [41] contrasted this with the much lower rate of individuals deciding on their own to seek services (16%). Families were found to be highly involved at various points along the pathway to care by recommending sources of help [28, 52], being the most common first source of help [36, 39, 62], directly initiating contact [31, 41, 54] or being the most common contact [37, 60]. Studies’ methodologies may have influenced their findings. For example, while 12 studies included families/relatives in their definitions of help-seeking contacts, 26 studies only considered professional contacts. Seven studies did not explicitly describe their inclusion criteria for pathway contacts.

### Negative pathways to care

Negative pathways, generally defined as those involving contacts with the criminal justice system, emergency or inpatient units, are associated with poor patient experiences and disengagement [12]; and high costs, despite sometimes resulting in reduced treatment delays.

A number of studies explored the involvement of police and emergency services along pathways to care. In a study whose entire sample was African–American [34], over a quarter of participants had at least one contact with police, and police accounted for a fifth of all contacts. In another US study [33], the pathways of over half the Black participants featured some police involvement, a rate significantly higher than that observed in other ethnicities. In a Canadian study [24], emergency rooms were four and three times more likely to be the first contact for Asians and other ethnicities, respectively, than for White and Black participants. Overall, emergency services figured prominently as pathway agents across studies and contexts (*n* = 15).

### Costs

Two studies [30, 44] examined the costs associated with various pathways to care. In a Canadian study [30], pathways to care involving inpatient units were 18.5 times costlier than pathways with no inpatient unit involvement. This was attributable to the greater involvement of police and emergency services with participants who ended up being inpatients. An Indian study demonstrated that the median monetary cost of an individual’s pathway to care was more than half the average family’s monthly income [44].

### Conceptual frameworks

The only three studies that explicitly described being guided by a framework [26, 41, 50] all used Goldberg and Huxley’s conceptual framework [69]. This framework proposes that mental health problems manifest at five levels (from in the community to among those in specialized care), with individuals’ advancement to subsequent levels being checked by selectively permeable filters that pertain to problem recognition (e.g., by general practitioners) and referral (e.g., to specialized care).

### Quality appraisal

The methodological quality of the studies was mixed (see Table 4 for quality scores). Six studies met over 75% of the quality appraisal criteria; 34 studies met 50–75% of the criteria; and five studies met under 50% of the criteria. Key limitations were insufficient reporting on sample size determination; low participation rates or inadequate differentiation between participants and non-participants; and non-standardized ascertainment of pathways to care.

**Table 4:** Quality appraisal scores

Study	Research question	Representativeness of participants	Non-participation rate	Adequacy of sample size	Adjustment for confounding factors	Definition of pathways to care	Ascertainment of pathways to care	Measurement of pathways to care	Method of ascertainment
Addington et al. [22]	+	∙	+	−	−	+	+	+	+
Anderson et al. [12]	+	+	+	−	+	+	+	+	+
Anderson et al. [23]	+	∙	+	−	+	+	+	+	+
Archie et al. [24]	+	+	∙	−	+	+	+	+	+
Bakare [25]	+	∙	−	−	∙	+	+	−	+
Bekele et al. [26]	+	−	−	−	+	+	∙	+	+
Bhui et al. [27]	+	∙	−	−	+	−	−	+	+
Chadda et al. [28]	+	∙	−	−	+	−	−	+	+
Chesney et al. [29]	+	∙	−	−	∙	+	+	−	+
Cheung et al. [30]	+	+	−	−	∙	+	+	+	+
Chiang et al. [31]	+	∙	∙	−	−	−	+	−	+
Chien and Compton [32]	+	+	−	−	+	+	+	−	+
Commander et al. [33]	+	+	+	−	∙	+	∙	−	+
Compton et al. [34]	+	−	−	−	+	+	+	+	+
Cougnard et al. [35]	+	∙	+	−	+	+	+	−	+
Del Vecchio et al. [36]	+	∙	−	−	+	+	+	+	+
Ehmann et al. [37]	+	∙	−	−	∙	+	+	+	+
Etheridge et al. [38]	+	−	∙	−	∙	−	∙	−	+
Fridgen al [39]	+	+	−	−	∙	+	−	∙	+
Fuchs and Steinert [40]	+	∙	+	−	∙	−	∙	−	+
Giasuddin et al. [41]	+	+	+	+	+	+	∙	+	+
Hastrup et al. [42]	+	+	+	−	+	−	−	−	+
Hodgekins et al. [43]	+	∙	−	−	∙	+	+	+	+
Jain et al. [44]	+	∙	+	−	∙	+	+	+	+
Judge et al. [45]	+	∙	−	−	∙	+	+	−	+
Kurihara et al. [46]	+	∙	+	−	∙	+	+	+	+
Lahariya et al. [47]	+	∙	+	−	∙	−	+	+	+
Lincoln et al. [48]	+	∙	+	−	+	−	∙	+	+
McMiller and Weisz [49]	+	∙	∙	−	−	+	∙	−	+
Mkize and Uys [50]	+	−	−	+	+	+	∙	+	+
Naqvi et al. [51]	+	+	−	−	−	−	∙	−	+
Neubauer et al. [52]	+	∙	+	−	+	+	+	−	+
Norman et al. [53]	+	∙	−	−	∙	+	+	+	+
O’Callaghan et al. [54]	+	∙	+	−	+	+	∙	−	+
Phillips et al. [55]	+	∙	∙	−	−	−	+	−	+
Platz et al. [56]	+	+	−	−	−	−	+	−	+
Reeler [57]	+	∙	−	−	−	+	∙	+	+
Reynolds et al. [58]	+	∙	∙	−	+	+	−	−	+
Sharifi et al. [59]	+	+	+	−	∙	+	+	−	+
Shin et al. [60]	+	∙	−	−	∙	+	∙	−	+
Stowkowy et al. [61]	+	∙	−	−	+	+	+	+	+
Subramaniam et al. [62]	+	∙	−	−	+	+	∙	−	+
Turner et al. [63]	+	∙	+	−	∙	+	∙	−	+
Graf von Reventlow et al. [64]	+	+	+	−	+	−	+	+	+
Wiltink et al. [65]	+	∙	∙	−	−	+	∙	−	+

−, Criterion not met; •, Criterion partially met; +, Criterion satisfied

## Discussion

Pathways to mental healthcare for youths tend to be complex, with multiple help-seeking contacts, and, sometimes, lengthy delays before appropriate care begins. Across many contexts, general practitioners played a prominent role in the help-seeking process. The role of primary care is notable given the international consensus that integrating mental health services within primary care is essential to address gaps in mental healthcare provision [70].

In our reviewed studies, primary care physicians were more frequently among the first help-seeking contacts than a ‘successful’ referral source. To be the first line of mental healthcare, primary care providers must be adequately trained to effectively detect problems, render support, initiate treatment, coordinate with all healthcare tiers, and refer appropriately.

Across settings, families played an influential role along pathways to care. This highlights the need for including families as pathway agents, something only few studies did. It also indicates that families need to be targeted in outreach efforts to reduce treatment delays for youths. Thus, giving due regard to families is important because familial involvement is known to mitigate the negative effects of and facilitate recovery from many mental illnesses [71].

Given the increasing rates of hospitalization and emergency visits among youths with mental health problems [72], and the high rates of emergency services involvement noted in our review, it is necessary to improve our understanding of the determinants of and trajectories to these endpoints that are associated with high personal and societal costs. Notably, the reviewed studies offer limited insights into what determines which youths follow these negative pathways, barring examinations of ethnicity as a determinant in the case of psychosis [23, 24, 33, 34].

Many of the factors leading to fragmented or difficult access to mental health services occur across age ranges. Studies assessing pathways to care in young children [73] and older adults [74] have also reported complex trajectories prior to obtaining services. Notably, however, many mental health systems have attributes that are known to disrupt care specifically for youth; chief among these being the transitions from child–adolescent to adult services [15]. These transitions, often rigid and poorly executed, can lead to disengagement from services and poor clinical outcomes. As such, it may be important for future research to prospectively assess pathways into and through services, and to pay specific attention to how transitions across mental health systems contribute to treatment delays and complicated pathways.

### Reconceptualising pathways to care beyond psychosis

This review reveals that knowledge on pathways to care in youth mental health is largely driven by first-episode psychosis literature. This is likely due to the field’s focus on reducing the DUP. Despite some disagreements on optimal treatment [75], there is enough consensus on care benchmarks for early psychosis researchers to clearly define ‘appropriate care’ and precisely delimit youths’ pathways thereto. Also, most early intervention programs for psychosis target age groups that match our review’s age-based selection criterion.

There is an evidence base for the adequacy of treatment for mental disorders other than psychosis [76]. Efforts to quantify treatment delays have also expanded to more disorders, with the adoption of DUI measures in bipolar [77], anxiety [78] and mood [79] disorders. Yet, specific inquiries into pathways to care across these disorders, at least with respect to youth-focused literature, remain limited, as does our understanding of the association between pathways to care and treatment delays.

The concept of appropriateness of pathway contacts warrants reflection. In early psychosis, contacts following the onset of frank psychotic symptoms that do not result in the commencement of psychosis-specific treatment can be viewed as missed opportunities for early intervention and prevention. More generally in youth, however, mental health symptom presentations are often transient and overlapping, and sometimes difficult to distinguish from developmentally normative behavioural or mood changes. It may therefore be difficult to establish an optimal ‘pathway to care’ in the broad field of youth mental health, and especially challenging to determine whether and when individuals reach an appropriate service. Two identical pathways may, in one case, reflect the appropriate use of a stepped-care model or, in another case, an inappropriately complex pathway. Moreover, even for similar problems, different individuals may have different optimal endpoints, based on available services, individual preferences, previous experiences, etc. Such complexities notwithstanding, studies on pathways to care can yield a greater understanding of how treatment gets delayed; and help identify the key agents involved in young peoples’ help-seeking processes and targets for outreach.

It has been argued that ‘one-stop’ multidisciplinary integrated youth services [13] can improve pathways to mental healthcare for young people. A central tenet of these services is the concept that ‘every door is the right door’. Such services aim to cater to youths with a range of needs (e.g., physical health, sexual health, mental health, housing, etc.) and types/severities of mental health problems. Examples of integrated youth services initiatives includes headspace in Australia [80], Jigsaw in Ireland [81], Youthspace in Birmingham, UK [82] and ACCESS Open Minds, Foundry and Youth Wellness Hubs Ontario in Canada [83–85].

Only one study in our review [43] focused on pathways to care at a cross-diagnostic service that addressed severe and complex mental health conditions. We strongly recommend that the transformation of youth mental healthcare, including the establishment of youth hubs within community settings, be accompanied by increasing study of pathways to this presumably desirable endpoint. Such research is pertinent given young people’s preferences for community-based settings for mental healthcare [86].

### Contextual sensitivity

Pathways to care are quite variable across geographies, reflecting differences in healthcare, social, and cultural contexts. Many studies reported the attributes of their healthcare systems that may have influenced pathways to care. Importantly, individuals contacted many providers before reaching even those services that had open referral systems. This is perhaps unsurprising, given that, at least in psychosis, service configuration alone does not appear to impact treatment delays [87]. This finding underscores the importance of early identification and outreach in reducing treatment delays [66] as rapid access to care depends not only on systemic factors, but also on such influencers of help-seeking such as stigma, mental health literacy, and awareness of available services [88, 89].

Notably too, some studies reported longer referral delays than help-seeking delays, suggesting that the delay in treatment was attributable more to the care system itself. One can therefore conclude that the effort to reduce treatment delays and simplify pathways has to be directed at both the help-seeking and the referral components of treatment delay.

The importance of primary care physicians prevailed in settings promoting ‘stepped care’ or general practitioner-gatekeeper models (e.g., Canada, Australia and Western Europe). Some contexts that allowed direct access to specialized care were likely to report self- or family-initiated referrals. In general, the role of general practitioners seems to be influenced by features of the healthcare system such as the availability and affordability of private or public mental health professionals.

Our review included studies from both low- and middle-income countries (LMICs) and high-income countries. With more than 80% of the world’s population, LMICs deploy less than 20% of the world’s mental health resources [90]. Often in LMICs, specialized care is inaccessible to many. These differences were reflected in our review. Certain LMIC-based studies described a difficulty in accessing formal mental healthcare, and cultural factors that influenced help-seeking (e.g., faith healers). More pathways to care research is needed in LMICs that have begun emphasising the integration of youth mental healthcare into existing community structures such as school, primary care, and community campaigns [91]. Such research can yield valuable insights on whether pathways to mental health care are simplified when addressed through larger public health promotion and development initiatives.

Notably, only four studies were from the United States, a country that otherwise generates volumes of mental health research. This suggests that interest in pathways to care may itself be a feature of public healthcare systems.

Studies on pathways to care need to better report on the organization of local mental health services/systems, and beliefs about illnesses and services. This would help contextualize the appropriateness of potential routes to care across contexts.

### Measuring pathways to care

Many challenges remain in the assessment of pathways to care. The lack of standardization in the measurement of pathways to care is a major limitation that, in psychosis research, has been identified for over a decade [16].

Wide variance in the definitions of start- and endpoints of pathways; and what and who constitutes a help-seeking contact limits our ability to compare results across studies. In many cases, the instruments chosen to assess pathways to care had a major influence on findings. Studies varied in their inclusion of formal, informal and ‘novel’ (e.g., web-based) contacts. The only study that specifically probed it, found that the internet figured prominently in the help-seeking process.

Only three studies mentioned being guided by a theoretical framework, despite the frameworks for help-seeking behaviour and service use being available since the early 1990s [92, 93] and having been modified for mental healthcare pathways research.

Studies on pathways to care are often premised on assumptions about the desirability of fewer contacts and, less frequently, the undesirability of certain types of contacts. Most studies are descriptive and provide estimates of individual and aggregate numbers and types of contacts made before a defined endpoint. However, evidence is lacking for whether more contacts along the pathway necessarily translate into longer treatment delays. Factors other than simply the number and type of contacts (e.g., waitlists, multiple encounters with the same contact, multiple contacts ending in evaluation but no treatment, etc.) may have a greater impact on treatment delays. Furthermore, reports of the numbers and types of help-seeking contacts do not reveal whether different services were accessed concurrently; whether appropriate treatments or referrals were offered and declined; or whether contacts met the individual’s needs. Also, notably absent is any measurement of how youths themselves perceived various help-seeking contacts.

To advance research on pathways to youth mental healthcare and, thereby, youth mental health outcomes, we outline some key recommendations informed by our review. An important first step is standardization in the reporting of pathways to care. Specific recommendations in this regard are:

- Making it a standard to use and report theoretical frameworks in pathways to care research would facilitate better comparability across studies, more meaningful syntheses of extant knowledge, and easier identification of gaps.
- Studies on pathways to care should define pathways clearly, specifying start and endpoints.
- Studies should describe their intended methods of assessing pathways to care, justifying the choice of methodology in relation to study aims and the chosen theoretical framework. Ideally, an instrument with established psychometric properties should be used. Where a novel instrument is used, its psychometric properties must be established and/or described.
- The instruments should use a clearly specified timeframe, and techniques such as anchor dates should be employed to reduce the effects of telescoping bias, whereby events are recalled as occurring earlier or more recently than they actually did [94]. This will allow for the accurate estimation of treatment delay indices.
- Studies should report on whether specific types of contacts were defined a priori or post hoc after collecting personal narratives, and whether specific types of contacts such as informal contacts (e.g., friends) and online resources were probed for in the interview.
- Studies should describe key features of the healthcare context (e.g., universal healthcare, access based on insurance, etc.) and referral system (e.g., walk-in access; need for a referral from a general practitioner, etc.) of their study setting.

The emergence of integrated youth services that, across geographic contexts, strive to adhere to common principles [95] provides both a framework and an impetus for standardising the measurement of pathways to care. In addition to addressing the considerations for reporting of pathways to care outlined above, a standardized measure for pathways to care to be used across youth services should be relevant to and feasible for implementation in a range of contexts (urban, rural, Indigenous, high- or low-income, etc.). An ideal measure would capture pathways into the service (e.g., walk-in, referral, etc.); what was offered at the service (e.g., evaluation, short- or long-term treatment, crisis intervention, etc.); and pathways out of the service.

Integrated youth services aim to offer well-publicised, rapidly accessible entry into a range of services and supports (not only those pertaining to mental health). The implicit assumption that such broad-spectrum services translate into more direct pathways and shorter delays to appropriate mental healthcare needs empirical testing. Some integrated youth services only offer interventions to those with mild to moderate mental and substance use concerns, referring more complex cases to external services. Future research therefore needs to examine whether such integrated youth services also succeed in simplifying pathways to care for youth with complex presentations.

A foundational principle of current endeavours to transform youth mental healthcare has been a commitment to making services youth-oriented, and engaging youths in service design and evaluation. Consistent with this, the creation or deployment of any standardized measure of pathways to care should be conducted in partnership with youths and their families, and should pay due regard to youths’ perceptions of their pathways into care. Future studies would also do well to enquire about e-pathways to care, as youths are known to turn to the internet and social media in seeking mental health help [96].

### Limitations

Our potential for comparisons across contexts and populations was limited by the lack of a standard methodology for ascertaining and reporting pathways to care. Our review’s scope was shaped by its inclusion of only quantitative studies that tend to focus on numbers and types of help-seeking contacts. Other significant aspects of the help-seeking process, such as beliefs about illnesses, and perceived barriers and facilitators to help-seeking, are largely found in qualitative analyses of pathways to care. Quantitative and qualitative approaches can have complementary potentialities in pathways to care research [97]. Our age-based criterion was deliberately broad to accommodate studies that may have included, but not solely focused on, youths. However, this impedes our confidence in the applicability of our findings to exclusively youth-focused settings.

## Conclusion

Across contexts, young people’s pathways to mental healthcare are often complex and involve various formal and informal agents. Further research is necessary to better understand, and ultimately, to simplify and streamline pathways to appropriate services. This is an essential step towards ensuring easier, timelier access to care and, thereby, shaping youth mental health outcomes. More research is needed to address critical gaps in our knowledge of young people’s pathways to care for problems other than psychosis; the determinants of pathways; and the help-seeking behaviours of and service responses to underserved groups such as Indigenous youths, youth in protection/welfare systems, and homeless youths.

## Electronic supplementary material

Below is the link to the electronic supplementary material.

Search strategy, MEDLINE (DOCX 64 KB)

Quality appraisal tool (DOCX 87 KB)
