# Supplementary material for: Pathways to mental health services for young people: a systematic review
Source: Soc Psychiatry Psychiatr Epidemiol. 2018 Aug 22;53(10):1005–38. doi: 10.1007/s00127-018-1578-y (PMC6182505; doi:10.1007/s00127-018-1578-y)
Supplement: Supplementary file 1 — Search strategy, MEDLINE (DOCX 64 KB) [file 127_2018_1578_MOESM1_ESM.docx]

**Search strategy (MEDLINE)**

| exp Mental Disorders/ or exp Mental Health Services/ or exp Community Mental Health Services/ or exp Mental Health/ or mental health services.mp. or mental illness.mp. or mental disorder*.mp. |
| --- |
| AND((pathway* adj3 care) or (pathway* adj4 mental) or (pathway* adj3 psyc*) or (pathway* adj4 service*) or(pathway* adj3 health) or (pathway* adj4 model) or (pathway*adj3 referral*) or (pathway* adj3 help) or(pathway* ajd4 contact*) or (help-seeking adj3 contact*) or (help-seeking adj4 model) or (healthcare adj3contact*) or (help seeking adj3 experienc*) or (help-seeking adj3 step*) or (help-seeking adj3 pattern*) or (help-seeking adj3 delay) or (help-seeking adj3 duration) or (referral* ajd2 pattern*) or (contact* adj3 service*)or (contact* adj3 professional) or (help-seeking adj4 service) or (Health adj3 contact*) or (system adj3 delay*)or- (systemic adj3 delay*) or (referral adj3 delay*) or (treatment adj4 delay*) or (mental health service ajd3delay) or (care adj3 delay) or (navigator) or (journey of care) or (care contact*) or (point* of entry) or (entryadj2 care) or (entry adj2 service*)).mp |
