# Supplementary material for: Pathways to mental health services for young people: a systematic review
Source: Soc Psychiatry Psychiatr Epidemiol. 2018 Aug 22;53(10):1005–38. doi: 10.1007/s00127-018-1578-y (PMC6182505; doi:10.1007/s00127-018-1578-y)
Supplement: Supplementary file 2 — Quality appraisal tool (DOCX 87 KB) [file 127_2018_1578_MOESM2_ESM.docx]

Online Supplementary Material II : Quality Appraisal Tool

| **Rating criterion** | |
| --- | --- |
| 1. Was the research question clearly defined? | - No |
|  | - Yes |
| 2. Representativeness of participants | - No description of the derivation of the sample |
|  | - Somewhat representative (clinical sample) |
|  | - Truly representative (e.g, catchment area) |
| 3. Non-participation rate | - High rate and no description of differences OR non-participation not described |
|  | - High rate and differences described |
|  | - Low rate and differences described |
| 4. Adequacy of sample size | - No power calculation or inadequate sample to detect differences |
|  | - Authors demonstrate the sample was powered to detect differences |
| 5. Adjustment for confounding factors | - None |
|  | - Age and/or gender only |
|  | - Other risk factors to delays/pathways to care included |
| 6. Definition of pathways to care | - Definition of pathways to care unclear (e.g., no description of start/endpoints, types of contacts) |
|  | - Clear definition of pathways to care |
| 7. Ascertainment of pathways to care | - Not described / Chart review or third party only |
|  | - Patient report only |
|  | - Patient report corroborated with chart review or third party |
| 8. Measurement of pathways to care | - Not described / Non-systematic methods used for measuring pathways to care |
|  | - Use of standardized tool for measuring pathways to care |
| 9. Same method of ascertainment for entire sample? | - No |
|  | - Yes |
